# Brain Dynamics Underlying Reaching Movements Under Uncertainty

**DOI:** 10.1002/hbm.70643

**Published:** 2026-10-01

**Authors:** Nan Liu, Adam Zabicki, Gereon R. Fink, Paola Mengotti, Simone Vossel

**Affiliations:** ^1^ Cognitive Neuroscience, Institute of Neuroscience & Medicine (INM‐3), Forschungszentrum Jülich Jülich Germany; ^2^ Department of Psychology, Faculty of Human Sciences University of Cologne Cologne Germany; ^3^ Department of Neurology University Hospital Frankfurt Frankfurt am Main Germany; ^4^ Department of Neurology, Faculty of Medicine and University Hospital Cologne University of Cologne Cologne Germany

## Abstract

Motor responses are influenced by inferred predictability, but it remains unclear how uncertainty, or its inverse, precision, is encoded within motor control networks. In this study, we combined computational modeling of reaching movement response speed with functional magnetic resonance imaging (fMRI). We used a cueing paradigm with changing cue predictability (~50%, ~70%, and ~90%) to test how predictive inference about left/right reaching responses affected cortical activity and network connectivity in 28 healthy young adults. Behavioral results showed that the response speed differences between invalid and valid cueing increased with a higher model‐derived probability that the cue will be valid. The fMRI results revealed that activity in the bilateral intraparietal sulcus (IPS), the caudal part of dorsal premotor cortex (cPMd), and the cerebellum increased with higher uncertainty during valid trials. Moreover, the activation in the premotor cortex, bilateral inferior parietal lobe (IPL), IPS, and temporoparietal cortex was enhanced when reaching to invalidly cued targets (i.e., during motor reprogramming). Dynamic causal modeling revealed that uncertainty increased the connectivity from left to right IPS, from left IPS to cPMd, and from cPMd to primary motor cortex (M1) during valid trials. Motor reprogramming during invalid trials increased the coupling between bilateral IPL and strengthened pathways from IPL to M1 and from IPS to PMd, while decreasing connectivity from right to left IPS. These findings provide the first evidence of uncertainty‐dependent network dynamics during reaching movements in the human brain and shed light on distinct neural mechanisms underlying precision‐dependent motor control and motor reprogramming, with potential implications for psychiatric and neurological disorders characterized by aberrant precision control.

## Graphical abstract

Combining computational modeling, fMRI, and dynamic causal modeling, we reveal two distinct functional subnetworks within the frontoparietal motor network during reaching: an uncertainty‐sensitive network comprising the intraparietal sulcus, caudal dorsal premotor cortex, and primary motor cortex, and a reprogramming network involving inferior parietal and motor execution areas.

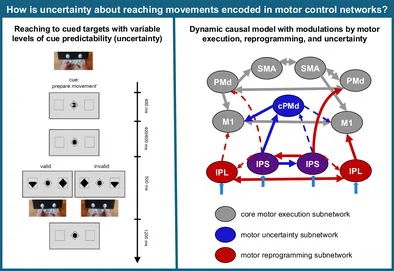

## 1 Introduction

Motor control is essential for performing most of our daily activities, such as talking, walking, and reaching for objects. The study of how individuals make accurate goal‐directed movements can have significant benefits in sports and medicine. Efficient motor control relies on predicting upcoming motor responses to external stimuli and updating (reprogramming) those predictions when the context changes. For example, when playing football, players constantly anticipate movements based on the ball's trajectory or teammates' positions. When unexpected events occur, such as a sudden interception or a rapid change in direction, they must quickly adjust their actions. This ability to prepare and reprogram motor responses, guided by prior information and learning from prediction errors, is fundamental for fast and flexible interactions with the environment. Therefore, the generation and updating of predictions (i.e., inferring the probability of events from observations) are essential for motor control.

Previous studies have investigated how probabilistic inference affects attention and motor preparation by adapting tasks in which cues precede behaviorally relevant targets (Dombert et al. 2016; Kuhns et al. 2017; Mengotti et al. 2022; Vossel et al. 2015). In these cueing tasks, the probability that a cue is valid (i.e., indicates the correct target location or motor response) is manipulated across levels, resulting in varying degrees of uncertainty. Evidence suggests that these unsignaled changes in cue predictability during the task affect reaction times (RTs), indicating that participants continuously infer the cue predictability in a given trial based on trial history. This inference process can be described by computational models such as the Hierarchical Gaussian Filter (HGF) (Mathys et al. 2011; Vossel et al. 2014).

A study applying the HGF model to RTs and including model‐derived cue predictability in the analysis of functional magnetic resonance imaging (fMRI) data showed that the blood‐oxygen‐level‐dependent (BOLD) response in the left angular gyrus and anterior cingulate cortex increased when a cue believed to be valid with a high probability turned out to be invalid (Kuhns et al. 2017). Using a similar motor cueing task with varying levels of cue predictability, Bestmann et al. (2008) demonstrated that corticospinal excitability—a measure of activity in the action preparation system—varied depending on the level of uncertainty associated with the upcoming action. Another study, comparing blocks in which the responding hand was known with blocks in which it varied randomly, showed that the random blocks were associated with heightened activity in parietal and motor areas during the prediction of upcoming actions (Jakobs et al. 2009). Still, it remains unclear how inferred trial‐wise predictability affects the connectivity within motor control networks in the human brain, motivating further investigations into the specific mechanisms of uncertainty‐dependent motor control.

Uncertainty may potentially be inferred and encoded within the parietal‐premotor network, including the posterior parietal cortex (PPC), the supplementary motor area (SMA), and the premotor cortex (PM). This network is responsible for movement intention and motor reprogramming (Cisek and Kalaska 2005; Dekleva et al. 2016; Desmurget and Sirigu 2009, 2012). A series of fMRI studies in humans has also shown that the prediction of events activates the motor system, particularly PM and its projections to parietal areas (Schubotz 2007; Schubotz and von Cramon 2002, 2003). Moreover, a recent transcranial magnetic stimulation (TMS) study applied stimulation either at the early or later stages of the occluder onset in an action video. The results revealed that early TMS over the left dorsal premotor cortex (PMd) elicited greater prediction errors than stimulation over the vertex, whereas late TMS had no effect. This suggests that PMd may play a role in the initiation of internally guided action prediction (Stadler et al. 2012).

The present study aimed to develop a comprehensive network model explaining how motor, premotor, and parietal brain regions interact to enable flexible control of reaching responses through inferred predictability, as well as motor reprogramming in invalid trials. To this end, we used computational modeling of response speed (RS) in a motor cueing paradigm with unsignaled changes in cue predictability (50%, 70%, or 90%) combined with fMRI and dynamic causal modeling (DCM) of different network architectures. We hypothesized that higher model‐derived cue predictability would accelerate responses in valid trials and slow responses in invalid trials, thereby increasing RS differences between the two trial types (i.e., the validity effect). Moreover, we expected that predictability/uncertainty modulates the activity of parietal areas and the effective connectivity between parietal and (pre)motor regions. In summary, hypotheses are based upon an active inference formulation of motor planning and execution. In these accounts of motor control as inference, predictions of the proprioceptive consequences of an intended movement are realized by motor reflexes (Adams et al. 2013; Feldman 2009; Todorov and Jordan 2002). These predictions are based upon hierarchical predictive coding driven by precision‐weighted prediction errors. Crucially, the precision—or inverse uncertainty—is itself predicted and instantiated through context‐sensitive changes in postsynaptic gain or excitability (Adams et al. 2013; Shipp 2016). In situations of uncertainty and unpredictability, the precision of high‐level (e.g., parietal) prediction errors is reduced, in relation to lower‐level (e.g., premotor) prediction errors. In engineering, this corresponds to adjusting the Kalman gain that figures in linear quadratic control (Rao 1999; Todorov 2008). Physiologically, the corresponding precision or postsynaptic gain control is generally thought to be mediated by neuromodulatory mechanisms of various sorts (Limanowski 2022; Pinotsis et al. 2014; Vossel et al. 2015). In terms of modeling, it is this balance of precision (i.e., inverse uncertainty) that is optimized by the HGF model. Neurophysiologically, we therefore hypothesized that—in valid trials—greater uncertainty would induce an increase in the precision and gain of neuronal populations at the lower levels of the sensorimotor hierarchy. This increase in excitability would then be measured as an increase in the effective connectivity of connections from a higher to lower cortical nodes. In invalid trials—that require a revision of attentional set—we hypothesized a similar change in descending connections in an extended system responsible for motor reprogramming. We used DCM to estimate these condition‐ and context‐sensitive changes in effective connectivity, in valid and invalid trials, respectively.

## 2 Materials and Methods

### 2.1 Participants

Based on the effect size from a similar cueing paradigm of a paired *t*‐test between the validity effect in a 90% and a 50% cue predictability condition (Kuhns et al. 2017; Cohen's *d* = 0.789), an a priori power analysis using G*Power 3.1 (Faul et al. 2007) indicated that at least 23 participants would be required to detect differences between these cue predictability conditions. To ensure a minimum sample size of 23 participants, accounting for drop‐outs, we recruited 30 healthy participants. All gave written informed consent before participating in the study and had normal or corrected‐to‐normal vision and no history of neurological or psychiatric conditions. Two participants had to be excluded from further analysis due to excessive head movement (framewise displacement > 2 mm during the task phases). Therefore, data from 28 participants were analyzed (13 females and 15 males; age ranging from 18 to 35 years; mean age: 25.1 years). All participants were right‐handed, as assessed by the Edinburgh Handedness Inventory (Oldfield 1971). This study was approved by the ethics committee of the German Psychological Society and performed following the Code of Ethics of the World Medical Association (Declaration of Helsinki).

### 2.2 Stimuli and Experimental Paradigm

Stimuli were displayed on a 30‐in. LCD screen (spatial resolution 1280 × 800, 60 Hz sampling rate) positioned behind the scanner. Participants viewed the monitor through an adjustable mirror mounted above the head coil.

A central diamond (2° wide) was presented on a gray background, and participants were asked to fixate it throughout the experiment (Figure 1A). Two boxes (2.5° wide and 5° eccentric in each visual field) on the left and right sides of the central diamond were displayed in each trial and could contain the target stimuli. Each trial began with the appearance of a cue (a white square) for 400 ms within either the left or right section of the central fixation diamond. This cue indicated the left or right target location and correspondingly a reaching movement of either the left or right hand. After a stimulus‐onset asynchrony (SOA) of 800 or 1000 ms, two diamonds were presented for 500 ms in the left and right boxes. The target diamond was the diamond with a missing upper corner. The distractor diamond was used to ensure comparable sensory input in both hemifields. Upon target presentation, participants were indeed required to release a response button and execute the movement, depending on the location of the target: they were asked to direct their index finger of their left hand toward left visual targets and their index finger of their right hand to right visual targets, touching a corresponding physical reaching target (ping pong balls resting on their abdomen). They were asked to move their wrist without moving their arm to reduce movement artifacts. Although the exact movement (wrist or arm) can influence neural activity patterns (Cavina‐Pratesi et al. 2018), similar movements (without touching) have been used as a proxy for reaching in previous work (e.g., Connolly et al. 2003; see Culham et al. 2006 for a review). The intertrial interval was 1200 ms. The photos in Figure 1A show the setup from a behavioral study with the same task. In the fMRI study, the button box and reaching targets were located on the participants' abdomen. Additionally, we set up an MRI‐compatible camera to record the participants' finger movements during the experiment. The videos were then reviewed to confirm that participants indeed performed the reaching movements (i.e., that they did not only release the response button without performing the movement to the target).

**FIGURE 1:**
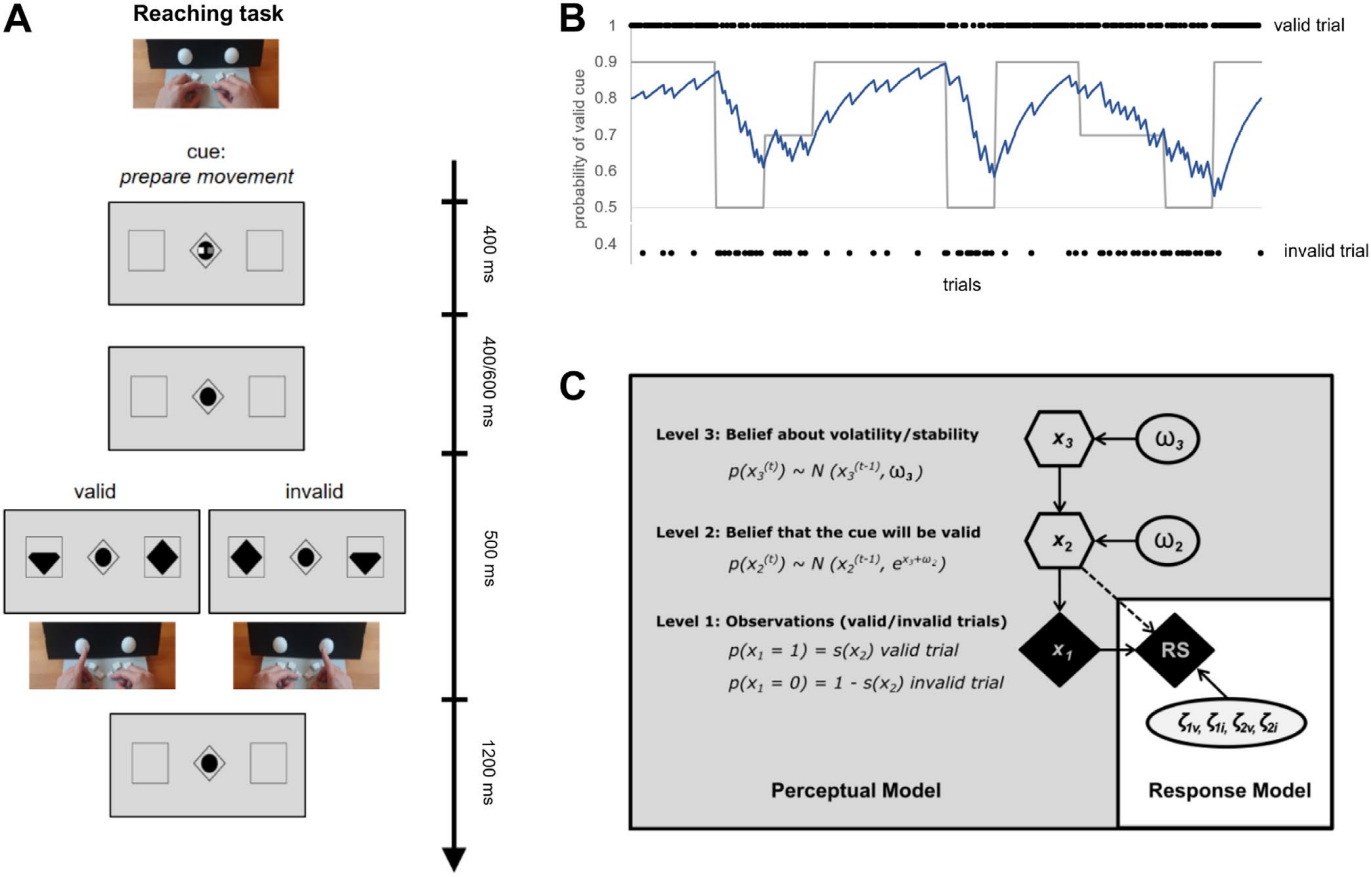
Experimental design and illustration of the Bayesian hierarchical model. (A) Illustration of the task and the setup from a behavioral study with the same reaching task. In the fMRI study, the button box and reaching targets were located on the participants' abdomen. (B) The probability of a valid cue was manipulated during the experiment, varying between ~50%, ~70%, and ~90% (gray line). A participant's trial‐by‐trial estimates of the probability that the cue will be valid, as derived from the Hierarchical Gaussian Filter (HGF) learning model, are shown as the blue line. (C) The Bayesian HGF model for trial‐wise belief updating. The perceptual model (shown on the dark gray background) incorporates the three states (*x*_1_, *x*_2_, *x*_3_). Higher levels are influenced by constant parameters *ω*_2_ and *ω*_3_, which affect trial‐wise changes at the respective level. Whereas the variables shown in diamonds and hexagons are quantities that evolve over time (trials), the circled variables are constants. Additionally, the quantities in the hexagons rely upon their previous states in a Markovian fashion.

Participants were instructed to use the cues to speed up their response and to respond to the target as quickly as possible. The cue could be either valid or invalid, that is, predicting the correct or incorrect target location and associated motor response. During the task, different proportions of valid and invalid trials were used to manipulate the uncertainty about the upcoming motor responses and target locations (Figure 1B). The cue predictability levels (%CP) were either ~50%, ~70%, or ~90% (Figure 1B, gray line). Participants were not informed about these specific levels or when they would change; they were only informed that %CP would vary throughout the experiment. In total, there were 217 validly cued and 67 invalidly cued trials. The number of left and right targets and the two cue‐target SOAs were approximately counterbalanced across valid and invalid trials. Moreover, 72 null trials, in which only the fixation diamond and the two peripheral boxes were displayed, were randomly intermixed with the experimental trials. In addition, 40 catch trials in which only the cue but no target appeared were included to prevent anticipatory responses before target presentation. In total, the fMRI experiment lasted for ~18 min, including two 30‐s rest periods. As a standard procedure in computational studies of trial‐by‐trial inference, each participant was presented with the same trial sequence (e.g., Iglesias et al. 2013). Before the task, participants performed a 5‐min practice session with a constant 80% cue predictability outside of the scanner. In addition to the reaching task, participants performed two other cueing tasks with different response modalities. The order of tasks was counterbalanced across participants, and a few training trials were presented before each task in the scanner as a reminder of the specific instructions. The present analyses focus solely on the reaching task.

### 2.3 MRI Data Acquisition and Preprocessing

T2*‐weighted echoplanar (EPI) images with BOLD contrast were acquired with a Center for Magnetic Resonance Research (CMRR) multiband sequence with a multiband factor of 8, a repetition time of 0.8 s, and an echo time of 0.037 s using a 3 T MRI system (Prisma, Siemens). A total of 1315 volumes with anterior‐to‐posterior (AP) phase encoding were acquired. In three subjects, the number of acquired volumes amounted to 1350 due to slightly longer inter‐trial intervals caused by a programming setting that provided online output within the Presentation software. Each volume consisted of 72 slices. The field of view was 208 mm with a 104 × 104 image matrix and a voxel size of 2 × 2 × 2 mm^3^. The first five volumes were discarded to allow for T1 equilibration effects. The remaining volumes were analyzed using the Statistical Parametric Mapping software SPM12 (Wellcome Department of Imaging Neuroscience, London; Friston et al. 1995; https://www.fil.ion.ucl.ac.uk/spm/). Additionally, 10 EPI volumes with a posterior‐to‐anterior (PA) phase encoding and a high‐resolution anatomical image (voxel size 0.9 × 0.9 × 0.9 mm^3^, T1‐weighted MPRAGE sequence) were acquired.

To ensure data processing quality, preprocessing and quality control were conducted according to a recently published protocol using SPM12 and MATLAB (Di and Biswal 2023). The single‐subject T1‐weighted image was first segmented and then spatially normalized to the MNI template, generating deformation fields. A skull‐stripped, bias‐corrected anatomical image was then generated for further analysis. The quality of segmentation was checked by overlaying the contour of the segmented gray matter density image onto the single‐subject T1‐weighted image in MNI space, as well as overlaying the segmented images of gray matter, white matter, and cerebrospinal fluid (CSF) onto the segmented gray matter density image in MNI space. Time differences between slices in EPI image acquisition were corrected to the middle slices. To remove movement artifacts, the EPI images were spatially realigned to the first image and subsequently re‐realigned to the mean of all images. Based on the estimated rigid body transformation results, we calculated framewise displacement in translation and rotation. The field inhomogeneity was estimated between the acquired EPIs with reverse phase encoding and the realigned EPI images, and susceptibility artifacts in these images were corrected using a physical distortion model from HySCO (Ruthotto et al. 2013, http://www.diffusiontools.com/documentation/hysco.html) built in the ACID toolbox (http://www.diffusiontools.com/index.html). A mean EPI image was generated after correction and was coregistered to the skull‐stripped, bias‐corrected anatomical image, while all EPI images were kept aligned with the mean EPI image. EPI images were also bias‐corrected. The quality of the coregistration between the anatomical and functional images was assessed by overlaying a functional image onto the skull‐stripped anatomical image and checking whether the contours of the two images aligned well. The deformation obtained in the first step was then applied to the individual EPI images to transform them into standard MNI space and resample them to 2 × 2 × 2 mm^3^ voxels. After normalization, the spatial registrations were also checked. Finally, the normalized images were spatially smoothed using an 8‐mm full‐width half‐maximum Gaussian kernel.

### 2.4 Eye Movement Recording and Analysis

Eye movements were recorded from each participant using an MRI‐compatible EyeLink eye‐tracker (SR Research) at a sampling rate of 1000 Hz. Before starting the reaching task, 9‐point eye‐tracker calibration and validation were performed to identify and ensure the accurate gaze position of the right eye. Data were analyzed using MATLAB and custom scripts to ensure that the participants had maintained fixation during the cue period (from cue onset to target onset) and the target period (from target onset to offset). Only trials with less than 50% missing data points within the time interval of interest were included in the analyses. Fixation time was calculated as the percentage of the time interval of interest during which fixation was maintained within a circle centered on the central fixation point, with a radius corresponding to the size of the diamond cue.

### 2.5 Statistical Analysis of Behavioral Data

For catch trials, the percentage of false alarm responses was determined and was analyzed with a within‐subject analysis of variance (ANOVA) with the factor *true %CP* (50/70/90). For regular trials, RTs faster than 100 ms and RTs deviating by more than 3 standard deviations (SDs) from the individual mean were excluded from the RT analysis. Mean RTs and accuracy were first analyzed as a function of *validity* (valid/invalid) and *true %CP* (50/70/90) using a within‐subject ANOVA, followed by post hoc *t*‐tests with Bonferroni–Holm correction. Here, we hypothesized that the RT difference between valid and invalid trials (i.e., the cueing or validity effect) would increase with higher %CP, as reflected in a significant interaction effect. This would indicate that participants indeed inferred the different %CP levels and adapted their behavior accordingly, thereby justifying the application of the computational model in a subsequent step. Two additional ANOVAs were performed: one examining the effects of the side of the presented target/hand movement (*validity* (valid/invalid) × *true %CP* (50/70/90) × *side of target/hand* (left/right)); and the other one analyzing the effects of cue‐target SOA (*validity* (valid/invalid) × *true %CP* (50/70/90) × *SOA* (800/1000 ms)).

For further modeling analysis, we focused on inverse RTs (i.e., RSs), as RSs can help normalize the distribution and potentially reflect the rate of information processing. As in previous work, single‐trial RSs were used to estimate, in each participant, trial‐by‐trial beliefs about cue predictability (i.e., the probability that the cue will be valid) using a hierarchical Bayesian observer model (HGF; Mathys et al. 2011). The HGF perceptual (learning) model consists of hierarchically coupled Gaussian random walks that enable a flexible updating of beliefs about cue predictability in each trial in relation to beliefs about volatility and subject‐specific parameters. It comprises three states denoted by *x*. The state $x_{1}^{\left(t\right)}$ represents the environmental state in each trial *t*, which, in the present paradigm, consisted of either a validly or invalidly cued target (with $x_{1}^{\left(t\right)}$ = 1 for valid and $x_{1}^{\left(t\right)}$ = 0 for invalid trials). The distribution of the probability of a trial being valid (i.e., $x_{1}^{\left(t\right)}$ = 1) is governed by the next higher state $x_{2}^{\left(t\right)}$ through a sigmoid (softmax) transformation. $x_{2}^{\left(t\right)}$ changes from trial to trial as a Gaussian random walk, determined by two quantities: $x_{3}^{\left(t\right)}$ (the state of the next upper level of the hierarchy) and a subject‐specific updating parameter *ω*_2_. The third state $x_{3}^{\left(t\right)}$ represents the belief about the stability of cue predictability, and also changes as a Gaussian random walk, with the step size determined by a second subject‐specific parameter *ω*_3_.

To infer the individual estimated trial‐by‐trial cue predictability from the RSs, the perceptual model needs to be inverted; this yields the posterior densities over the three hidden states $x^{\left(t\right)}.$ In the following, the means of the subject's posterior beliefs are denoted by $\mu^{\left(t\right)}$. Variational model inversion under a mean field approximation yields simple analytical update equations—where belief updating rests on weighted prediction errors according to an individualized approximate Bayes‐optimality, with reference to the subject‐specific values for the updating parameters *ω*_2_ and *ω*_3_ (Mathys et al. 2011). In this study, they provide the model‐derived cue predictability/uncertainty (i.e., the subject's estimate of the probability that the target requires the cued reaching response in a given trial).

The model inversion requires a response model, which describes how these beliefs translate into observable behavior. Consistent with previous work (Dombert et al. 2016; Kuhns et al. 2017), we used a response model in which RS is a linear function of the estimated probability that the cue will be valid ${\hat{\mu }}_{1}^{\left(t\right)}$. ${\hat{\mu }}_{1}^{\left(t\right)}$ is the estimated cue predictability before the observation of the outcome in trial *t*, and is derived from a sigmoid transformation of the value of $\mu_{2}^{\left(t-1\right)}$ from the previous trial. Whereas high probability estimates are expected to result in faster responses (higher RS) in valid trials, responses should be slower with higher estimated cue predictability in invalid trials (i.e., when invalid trials are less expected). Two response model parameters $\zeta_{1}$ and $\zeta_{2}$ parameterize the intercept and the slope of the linear function for valid and invalid trials, respectively:

$${\mathrm{RS}}^{\left(t\right)}=\left\{\begin{array}{c} \zeta_{1v}+\zeta_{2v}{\hat{\mu }}_{1}^{\left(t\right)}\;\text{for}\;x_{1}^{\left(t\right)}=1\;\left(i.e.,\text{valid trial}\right)\\ \;\zeta_{1i}+\zeta_{2i}\left(1-{\hat{\mu }}_{1}^{\left(t\right)}\right)\;\text{for}\;x_{1}^{\left(t\right)}=0\;\left(i.e.,\text{invalid trial}\right) \end{array}\right.$$

The perceptual and response models are sometimes also referred to as the “subjective” and “objective” models, respectively. The subject‐specific parameters *ω*_2_ and *ω*_3_ of the perceptual (subjective) model and the parameters of the response (objective) model were estimated using a variational Bayesian estimation, as implemented in the HGF as part of the TAPAS toolbox (version 7.0; Frässle et al. 2021; https://www.translationalneuromodeling.org/tapas). The individual trial‐wise estimates of cue predictability/uncertainty ${\hat{\mu }}_{1}^{\left(t\right)}$ were subsequently derived and entered into the analyses of the fMRI data (see the next section). Additionally, a 2 (*validity*: valid/invalid) × 4 (${\hat{\mu }}_{1}^{\left(t\right)}$: 0.64/0.76/0.88/1.00, averaged across 0.12 bins) within‐subject ANOVA on mean RSs was performed.

### 2.6 Statistical Analysis of Imaging Data

The fMRI data were analyzed using a random‐effects general linear model (GLM). At the single‐subject level, four experimental conditions were modeled: valid trials for left and right targets, and invalid trials for left and right targets. Each condition was modeled by two regressors: a main regressor coding event onsets as delta functions of constant amplitude, and a parametric regressor in which the amplitude of each event was scaled by the trial‐wise estimated cue predictability ${\hat{\mu }}_{1}^{\left(t\right)}.$ The parametric expansion was first‐order, and parametric regressors were mean‐centered with respect to their corresponding main regressor, such that the main regressors captured the mean evoked response per condition and the parametric regressors captured the component of the response scaling with ${\hat{\mu }}_{1}^{\left(t\right)}$ (i.e., the trial‐wise influence of uncertainty about the target location and motor response). Catch trials, in which participants responded correctly, were modeled separately. In addition, the two resting periods and error trials (incorrect or missing responses in regular trials and false alarm responses in catch trials) were modeled separately as further regressors of no interest. The six movement parameters of the rigid‐body realignment, along with their squared values, were included in the design matrix as additional nuisance regressors. We confirmed that framewise displacement did not differ between valid and invalid trials, and details are provided in the Supporting Information. All regressors of interest were convolved with the canonical hemodynamic response function (HRF) implemented in SPM12, without temporal derivatives. Since the cue–target interval was either 800 or 1000 ms, cue‐ and target‐locked regressors are highly correlated after convolution with the HRF. We therefore time‐locked events to target onset and did not model the cue as a separate event. Data were high‐pass filtered at 1/128 Hz. For each subject, 8 condition‐specific contrast images were created (main regressor and parametric modulator for each trial type).

At the group level, contrast images were analyzed using an eight‐condition GLM that included both the four main HRF regressors (2 [*validity*: valid/invalid] × 2 [*target location*: left/right]) and the four parametric regressors. We tested for the effect of target location to identify regions involved in left hand/leftward versus right hand/rightward reaching movements with two *t*‐contrasts. For the parametric regressors, we tested for negative parametric effects with cue predictability in valid trials and positive parametric effects in invalid trials (in analogy to their degree of unexpectedness and to the behavioral modulations by cue predictability). The *t*‐contrast of all invalid > valid trials was used to isolate brain regions involved in motor reprogramming. All contrasts were thresholded at *p* < 0.05 familywise error, whole‐brain corrected at the cluster‐level (with a voxel‐level cutoff of *p* < 0.001).

### 2.7 Region of Interest Definition

The aim of this study was to understand how inferred predictability is encoded within motor control networks. Therefore, based on the GLM results, we selected coordinates for DCM analyses within brain regions involved in motor execution per se, in the processing of uncertainty in valid trials, and in motor reprogramming in invalid trials, respectively.

Bilateral precentral gyrus (M1), PMd, and SMA were defined as the core motor execution subnetwork based on two criteria: peak voxels were selected using the activation map from a group‐level *F*‐contrast of valid_left versus valid_right trials (thresholded at the voxel‐level of *p* < 0.001 (uncorrected), see Table S1); inclusively masked by corresponding anatomical motor regions derived from the JuBrain Anatomy Toolbox (Eickhoff et al. 2005; https://github.com/inm7/jubrain‐anatomy‐toolbox). Similarly, the bilateral inferior parietal lobule (IPL) was functionally selected based on the group‐level *t*‐contrast of invalid > valid trials (thresholded at the voxel‐level of *p* < 0.001, uncorrected), and anatomically masked by areas PF and PFm of the JuBrain Anatomy Toolbox. Based on the group‐level GLM results, the bilateral intraparietal sulcus (IPS) was involved in both motor reprogramming during invalid trials and uncertainty modulation during valid trials. Accordingly, the bilateral IPS was defined by significant activation from the negative modulatory effect of estimated cue predictability in valid trials (thresholded at the voxel‐level of *p* < 0.05, uncorrected), and then masked by a group‐level *t*‐contrast of invalid and valid trials using the same threshold and by an anatomical IPS template of joint IPS subdivisions defined in the JuBrain Anatomy Toolbox. For the ROI involved in uncertainty processing, we selected the caudal part of PMd (cPMd)—specifically cytoarchitectonic area 6d1 (Ruland et al. 2025)—using a subpeak of the cluster showing a negative modulatory effect of estimated cue predictability in valid trials (thresholded at the voxel‐level of *p* < 0.001, uncorrected). We chose this region of the cluster because the peak coordinate (−4, −36, 72) of the cluster was located more posteriorly in the postcentral gyrus, and previous evidence suggests that cPMd is more involved in motor uncertainty processing than the postcentral gyrus (Dekleva et al. 2016; Schubotz et al. 2003). Table S1 provides an overview of the coordinates for all regions of interest.

### 2.8 Time Series Extraction

Participant‐specific time series were extracted from the nearest local maximum within a radius of 8 mm from the group maximum within specific ROIs that were selected based on the group GLM analysis (Table S1), using a significance level of *p* < 0.1 (uncorrected at the individual subject level). The first eigenvariate was then computed across all voxels within 4 mm of the subject‐specific maximum, using an “effects of interest” *F*‐contrast to focus on BOLD responses that reflect the effect of the modulation of cue predictability and invalid and valid cueing.

### 2.9 Specification of DCMs

We specified standard bilinear deterministic DCMs (Friston et al. 2003) using SPM12. The fixed connectivity within the network (DCM A‐matrix), which was identical for all models, is shown in Figure 4A. For simplicity and to keep the model space tractable, we did not include visual areas processing the target stimuli or the cerebellar regions showing an uncertainty‐related modulation in our DCMs. Instead, driving inputs (valid and invalid trials, collapsed over left and right targets) entered bilateral IPS and IPL, because these parietal areas supposedly receive direct input from the visual areas. The DCM analysis focused on the condition‐specific modulation of connectivity between the different ROIs (B‐matrix) by three effects in each competing model according to our GLM results: (1) connectivity changes related to left versus right hand reaching movements in the core motor execution network; (2) connectivity changes due to the modulatory effect of model‐derived cue predictability/uncertainty during valid trials; and (3) connectivity changes during motor reprogramming in invalid trials. The connectivity among the core motor execution areas during left‐ and right‐hand movements was specified according to previous findings and theoretical frameworks (see Figure 4B, upper panel; Crawford et al. 2011; Michely et al. 2018; Pool et al. 2014), and was identical for all tested models. For the remaining modulatory effects, we employed a nested model comparison procedure based on model families. First, we specified three competing alternative architectures how uncertainty in valid trials may modulate the coupling between the regions identified in the GLM analyses (IPS and cPMd) and areas of the core motor execution subnetwork. Here, the modulatory effects concerned the connections from left and right IPS to cPMd, as well as connections from cPMd to either (1) M1; (2) PMd; or (3) both M1 and PMd (see Figure 4B, middle panel). Second, we specified six possible architectures representing different hypotheses about interregional connections modulated by motor reprogramming in invalid trials (see Figure 4B, lower panel). These six alternative reprogramming modulations were combined with each of the three different types of uncertainty modulation to create three different uncertainty‐related model families (i.e., a model space of 3 × 6 = 18 different models, each comprising the same hand movement modulation, as well as a specific configuration of the uncertainty and motor reprogramming modulation). For clarity, the different modulatory effects are illustrated separately in Figure 4B.

### 2.10 Model Selection and Parameter Inference

We elected to use a fixed‐effects model selection procedure—as opposed to random effects analysis with parametric empirical Bayes—to maximize the efficiency of our connectivity estimates and their context‐sensitive changes within the motor system. In other words, we were not interested in the between‐subject variability in these estimates. We compared the three model families using fixed‐effects Bayesian model selection (Penny et al. 2010; Stephan et al. 2010). Subsequently, we applied Bayesian model averaging (BMA) to the winning uncertainty modulation family to estimate the parameters for each connection of the modulatory effects. BMA weights the model‐specific parameter estimates by the probability of the model containing the parameter. The probability that each mean DCM parameter from the averaged winning family model was nonzero was computed from its posterior distribution, using the mean and SD estimated from the BMA results.

## 3 Results

### 3.1 Eye Movement Data

On average, there were 257.5 ± 39.3 (mean ± SEM) analyzable trials for eye movement analysis during the cue period, and 260.3 ± 37.7 trials during the target period per participant. Participants maintained central fixation in 98.0% ± 0.4% (mean ± SEM) of the time during the cue period and in 90.8% ± 2.3% of the time during the target period. Figure S1A shows the mean percentage of fixation time during the cue period and in valid and invalid trials during the target period as a function of true %CP.

### 3.2 Behavioral Data

The percentages of false alarm responses during the catch trials, in which no target was present and responses had to be withheld, were 2.98% ± 1.50% (mean ± SEM), 3.92% ± 1.39%, and 4.32% ± 0.79% for the 50%, 70%, and 90% true CP conditions. A one‐way ANOVA on these false alarm rates revealed no significant effect of *true %CP* (*F*_(1,27)_ = 0.31, *p* = 0.68, *η*ₚ^2^ = 0.01), despite the numerical trend across conditions.

Mean RTs and accuracy in the three true %CP levels are given in Table 1 and depicted in Figure 2A,B. A 2 (*validity*: valid/invalid) × 3 (*true %CP*: 50/70/90) within‐subject ANOVA on mean RTs yielded a main effect of *validity* (*F*_(1,27)_ = 42.69, *p* < 0.001, *η*ₚ^2^ = 0.61) and a main effect of *true %CP* (*F*_(2,54)_ = 3.99, *p* = 0.024, *η*ₚ^2^ = 0.13), indicating faster responses in valid compared with invalid trials as well as faster responses with higher %CP. As hypothesized, the *validity* × *true %CP* interaction effect was significant (*F*_(2,54)_ = 13.08, *p* < 0.001, *η*ₚ^2^ = 0.34), indicating a differential RT modulation by %CP in valid and invalid trials. Post hoc *t*‐tests with Bonferroni–Holm correction revealed that in valid trials, responses were significantly faster at 70% CP and 90% CP compared to 50% CP (*t*_(27)_ = −3.19, *p* = 0.018; *t*_(27)_ = −4.54, *p* < 0.001). Responses were also significantly faster at 90% CP than 70% CP (*t*_(27)_ = −2.97, *p* = 0.025), indicating a general trend of faster responses with higher %CP. However, in invalid trials, responses did not significantly differ between any of the three %CP levels (all *p* > 0.05), suggesting that the modulation effect of true %CP on RT was only observed in valid trials. The same ANOVA performed on accuracy revealed only a main effect of *validity* (*F*_(1,27)_ = 19.68, *p* < 0.001, *η*ₚ^2^ = 0.42), indicating that participants were more accurate in valid than invalid trials. Both the main effect of *true %CP* and the interaction were not significant (*F*_(2,54)_ = 1.01, *p* = 0.37, *η*ₚ^2^ = 0.04; *F*_(2,54)_ = 1.97, *p* = 0.15, *η*ₚ^2^ = 0.07). The effects of the *side of target location/han*d and *SOA* are presented in the Supporting Information and illustrated in Figure S1B–E. While the *target/hand side* did not show significant main or interaction effects, SOA affected the %CP modulation in invalid trials.

**TABLE 1:** Behavioral data.

	Validity	%CP		
		50	70	90
Mean RTs (ms)	Valid	425.9 (±11.4)	416.0 (±11.0)	406.8 (±9.2)
	Invalid	452.3 (±12.9)	454.3 (±12.3)	452.0 (±11.2)
Mean accuracy (%)	Valid	97.4 (±0.5)	98.3 (±0.4)	97.9 (±0.2)
	Invalid	95.5 (±0.7)	93.8 (±1.1)	93.1 (±1.6)

*Note:* The numbers in parenthesis represent standard errors of the mean (SEM).

**FIGURE 2:**
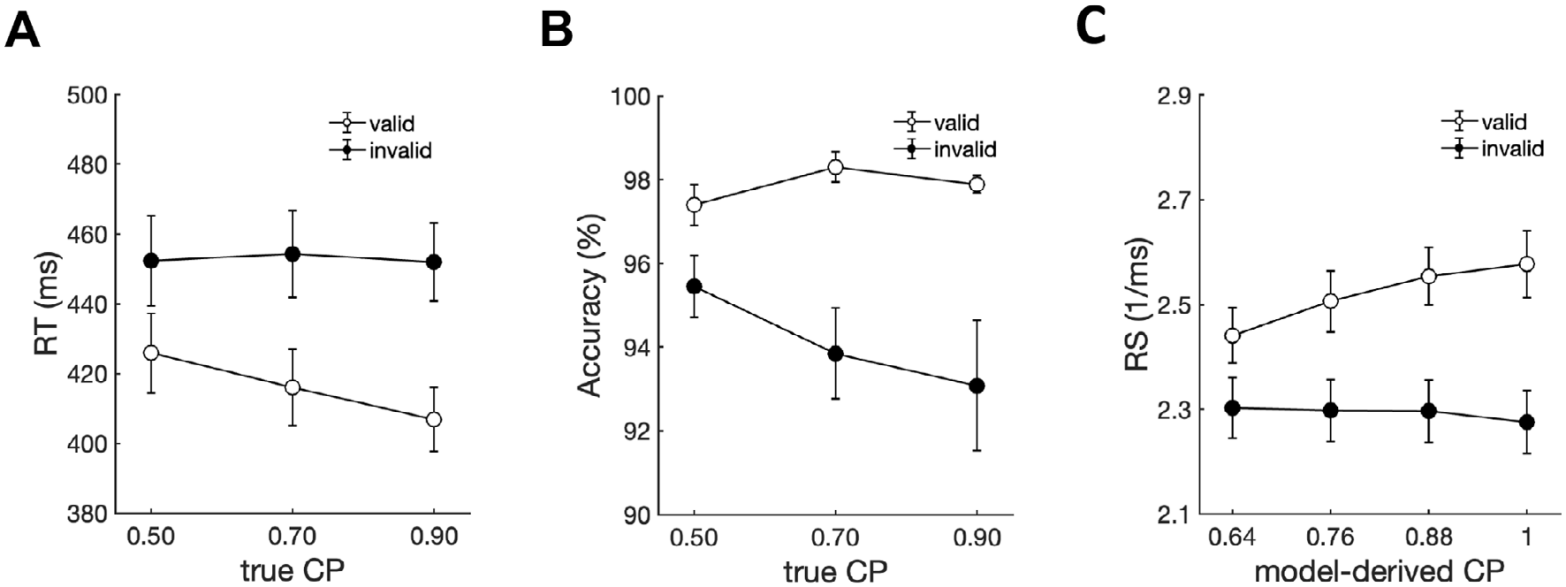
(A and B) Mean RT and accuracy in valid and invalid trials as a function of the experimentally manipulated CP level. (C) Mean RS for valid and invalid trials as a function of the binned HGF‐derived trial‐by‐trial estimate of cue predictability ${\hat{\mu }}_{1}^{\left(t\right)}$, which was calculated on the basis of group average values of the model parameters. Error bars represent SEM.

Next, subject‐specific trial‐by‐trial estimates of cue predictability ${\hat{\mu }}_{1}^{\left(t\right)}$ were derived from the HGF model (see Figure 1B for an example). The RSs in valid and invalid trials in relation to ${\hat{\mu }}_{1}^{\left(t\right)}$, averaged across 0.12 bins, are shown in Figure 2C. A 2 (*validity*: valid/invalid) × 4 (${\hat{\mu }}_{1}^{\left(t\right)}$: 0.64/0.76/0.88/1.00) within‐subject ANOVA on mean RSs revealed a significant main effect of *validity* (*F*_(1,25)_ = 59.68, *p* < 0.001, *η*ₚ^2^ = 0.71), again indicating faster responses to validly than to invalidly cued targets. The main effect of ${\hat{\mu }}_{1}^{\left(t\right)}$ was not significant (*F*_(3,75)_ = 2.68, *p* = 0.053, *η*ₚ^2^ = 0.10). The interaction of both factors was significant (*F*_(3,75)_ = 5.10, *p* = 0.003, *η*ₚ^2^ = 0.17), indicating a differential impact of cue predictability ${\hat{\mu }}_{1}^{\left(t\right)}$ in valid and invalid trials (Figure 2C). Post hoc *t*‐tests with Bonferroni–Holm correction showed that in valid trials, responses were significantly slower when the estimated cue predictability was in the lowest bin compared to the highest two bins (*t*_(27)_ = −3.29, *p* = 0.007; *t*_(27)_ = −3.65, *p* = 0.003), while in invalid trials responses did not significantly differ between any of the four levels of estimated cue predictability. Again, as in the model‐free RT results, the modulation was mainly present in valid trials.

### 3.3 Whole‐Brain GLM Analysis

To reveal the core motor regions involved in movement execution, we tested which brain areas were more active during reaching to left as compared to right targets with the corresponding left or right hand. The right > left contrast revealed activation in the left postcentral gyrus, M1, SMA, central opercular cortex, and the right cerebellum. The left > right contrast yielded activation of right motor areas, central opercular cortex, and the left cerebellum when reaching to left targets compared to right targets (Figure 3A and Table 2).

**FIGURE 3:**
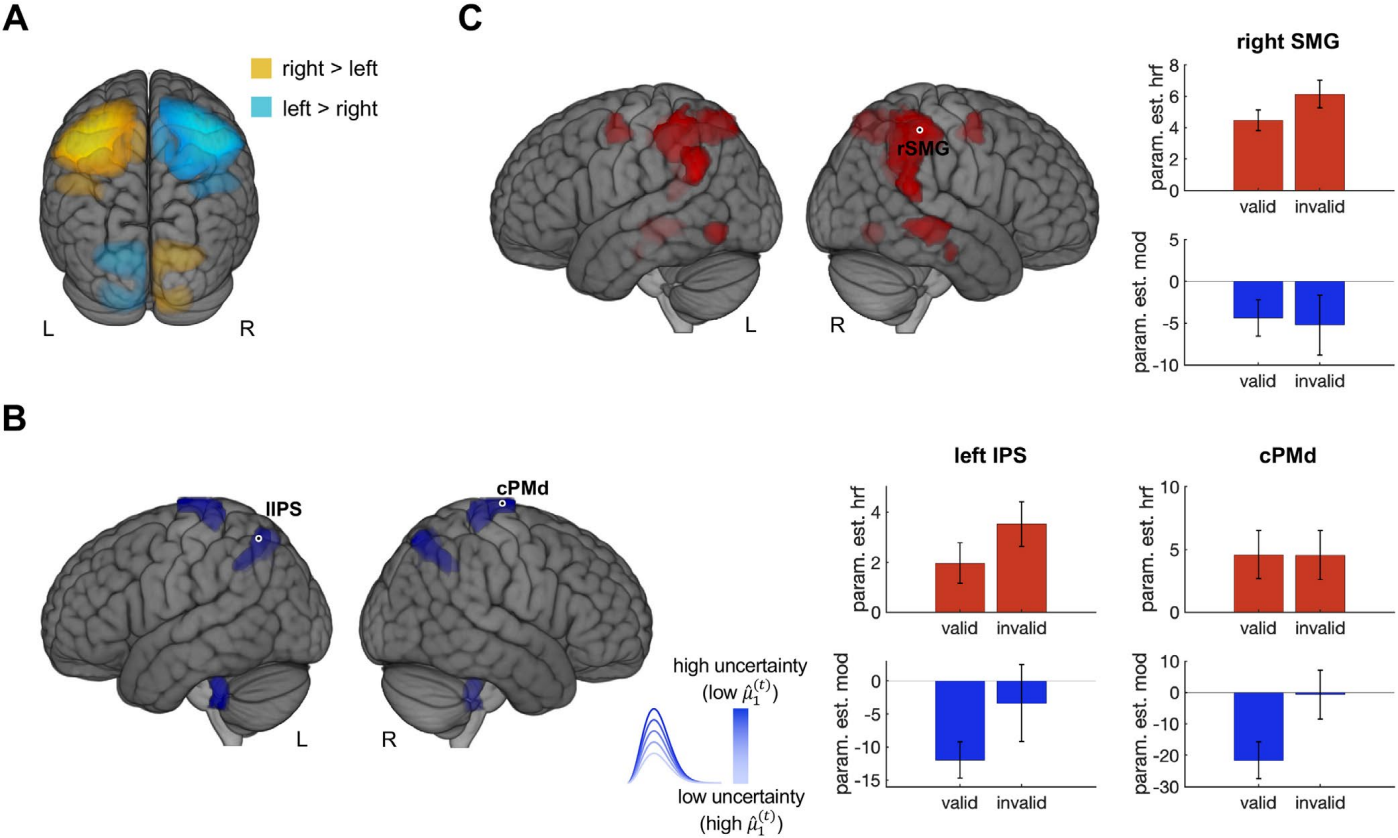
fMRI results of the GLM analysis. (A) Brain activations revealed by *t*‐contrasts of reaching to left versus right targets (*p*_FWEcl_ < 0.05). (B) Brain activations associated with a modulatory effect of model‐derived cue predictability ${\hat{\mu }}_{1}^{\left(t\right)}$ (uncertainty) on BOLD responses in valid trials (*p*_FWEcl_ < 0.05). The bar charts exemplarily depict the mean parameter estimates for the HRF regressor (upper, red) and for the parametric modulation with ${\hat{\mu }}_{1}^{\left(t\right)}$ (lower, blue) in valid and invalid trials in the left intraparietal sulcus (IPS) and caudal part of the dorsal premotor cortex (cPMd). Lower cue predictability in valid trials, indicating greater uncertainty about the target location, was associated with greater activity in bilateral IPS and cPMd. (C) Brain activations revealed by a *t*‐contrast of invalid versus valid trials for the HRF regressor (*p*_FWEcl_ < 0.05). The bar charts exemplarily depict the mean parameter estimates for the HRF regressor (upper) and for the parametric modulation with ${\hat{\mu }}_{1}^{\left(t\right)}$ (lower) in valid and invalid trials in the right supramarginal gyrus (SMG).

**TABLE 2:** Brain regions and their MNI coordinates identified in the GLM analysis.

Region	Side	MNI coordinates			Voxels	*Z*‐score
		*x*	*y*	*z*		
Reaching to right > left targets						
Postcentral gyrus	L	−44	−24	56	5522	21.3
M1	L	−38	−22	58		21
Cerebellum	R	20	−48	−22	2648	15.5
Central opercular cortex	L	−42	−22	18	1136	9.12
SMA	L	−6	−16	50	707	9.13
Reaching to left > right targets						
M1	R	34	−24	56	6738	20
SMA	R	10	−20	48		8.82
Cerebellum	L	−18	−50	−22	2776	14.7
Central opercular cortex	R	46	−18	18	1105	8.2
Negative modulatory effect of model‐derived cue predictability						
Postcentral gyrus	L	−4	−36	72	691	4.75
cPMd	R	2	−16	78		4.17
IPS	R	26	−70	56	377	4.33
	L	−26	−52	44	289	3.89
Cerebellum	L	−24	−38	−36	278	5.91
Invalid > valid trials						
SMG	R	44	−34	46	2669	5.22
IPS	R	32	−46	50		5.02
	L	−38	−44	48	2096	5.50
Middle temporal gyrus	R	54	−36	−8	724	4.98
Lateral occipital gyrus	L	−42	−64	−10	231	4.81
SMG	L	−56	−52	26	475	4.94
PMd	L	−24	−8	50	271	4.72
PMv	R	30	−2	50	215	3.91

Abbreviations: cPMd, caudal part of dorsal premotor cortex; IPS, intraparietal sulcus; M1, primary motor cortex; PMd, dorsal premotor cortex; PMv, ventral premotor cortex; SMA, supplementary motor area; SMG, supramarginal gyrus.

Regarding modulations of neural activity by uncertainty, we found a negative modulatory effect of HGF‐derived cue predictability ${\hat{\mu }}_{1}^{\left(t\right)}$ on BOLD responses in valid trials in a cluster comprising the left medial postcentral gyrus and cPMd, as well as in bilateral IPS and the left cerebellum (Figure 3B and Table 2). In these regions, activity increased with lower cue predictability (i.e., with more uncertainty about the required motor response) (Figure 3B). Similar to the behavioral pattern, no significant uncertainty modulations were observed in invalid trials. The contrast of invalidly versus validly cued targets (capturing brain regions involved in motor reprogramming, that is, the change of the prepared movement plan (the cued hand and target) when the target appears on the unexpected side) revealed increased activation in bilateral IPL, IPS, temporoparietal cortex, lateral occipital cortex, and PM in invalid trials (Figure 3C and Table 2). Interestingly, IPS showed significant effects in both contrasts (uncertainty modulation and reprogramming; see Figure 3B,C).

To further clarify the anatomical location of the different activated areas within subregions of PMd and other motor regions, and to distinguish the functional role of cPMd from other motor areas, we overlaid the activation in cPMd and in the left and right movement‐related areas onto the same brain template, and plotted the parameter estimates in the different experimental conditions (Figure S2). As depicted in Figure S2, cPMd was located more medially compared to the movement‐related areas in PMd, was modulated by uncertainty in valid trials, but showed no significant difference between reaching to left and right targets. In contrast, movement‐related motor areas (including M1, PMd, and SMA) that were activated during left/right reaching movements exhibited a significant contralateral movement effect but were not influenced by uncertainty.

### 3.4 DCM

One participant was excluded from the DCM group‐level analyses because this participant's DCM showed very low explained variance (1%) across all 18 models, indicating poor model fit. For the remaining 27 participants, we employed family‐level fixed‐effects Bayesian model selection to reveal the most likely connectivity architecture describing the connectivity modulation by uncertainty in valid trials (see Figure 4).

**FIGURE 4:**
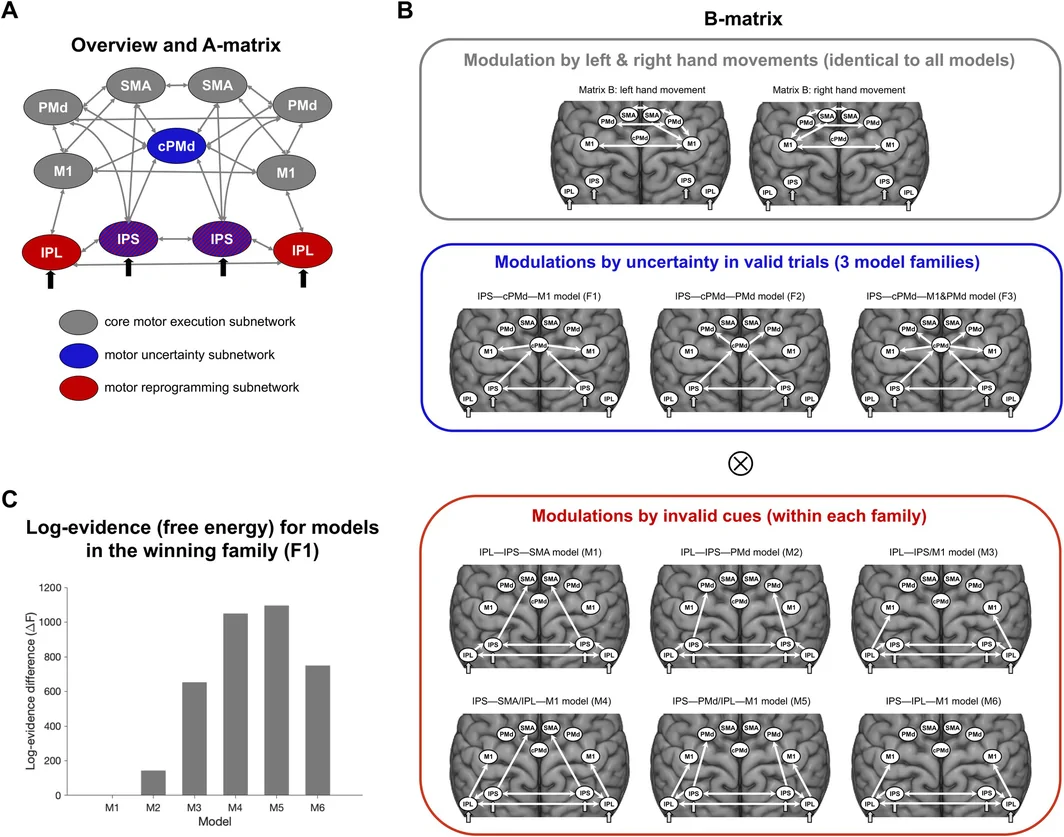
(A) Illustration of the composition of the network for the DCM analyses and the fixed connectivity (A‐matrix). The ROIs include areas involved in reaching movements (i.e., M1, PMd, and SMA), specifically the core motor execution subnetwork; bilateral IPL, which was involved in motor reprogramming; cPMd, modulated by motor uncertainty; and the bilateral IPS, which was involved in both motor reprogramming and uncertainty. (B) Illustration of the different condition‐specific modulatory effects (B‐matrix) and the construction of the model space for a nested family‐level Bayesian model selection. Note that the modulatory effects are presented separately in the figure for simplicity, but that each DCM comprised the hand movement modulation and a specific uncertainty and motor reprogramming modulation, yielding a total of 18 alternative comprehensive models. Left/right‐hand movement effects (upper panel) were identical for all models. Three hypothesized models for uncertainty modulation in valid trials (middle panel) were used to create three model families, each comprising six models with different modulations related to motor reprogramming in invalid trials (lower panel). The three model families were compared using fixed‐effects inference to identify the most likely family. (C) Within the winning family (F1), log‐evidence differences (ΔF) for the six models, computed under fixed‐effects inference as the group‐level variational free energy with the minimum subtracted (lowest‐evidence model is zero). Although Model 5 had the highest log‐evidence, Bayesian model averaging was performed across all six family members, as this is the principled Bayesian procedure for parameter inference under residual within‐family model uncertainty: Each model's estimates are weighted by its posterior probability, so a dominant model contributes most and weaker models are weighted lower. The resulting Bayesian model averaging estimates were correspondingly dominated by Model 5.

The results showed that the model family containing an uncertainty modulation of connections from cPMd to bilateral M1 (depicted in the left variant in Figure 4B, middle panel) was superior to other families, making it the winning family (family posterior probability = 1). Within the winning family, log‐evidence differences for the six models were computed under fixed‐effects inference as the group‐level variational free energy with the minimum subtracted (see Figure 4C). Next, BMA was applied to all six models of the winning family, each comprising the same uncertainty (as well as hand movement) modulation, but a different modulation for motor reprogramming in invalid trials (see Figure 4B, lower panel). Thereby, we estimated the parameter for each modulatory effect and connection by accounting for differences in the probability of the six different motor reprogramming models. Model 5 had the highest log‐evidence (Figure 4C) within the winning family. Its posterior weight in the BMA, therefore, dominated to numerical precision, so connections specified only in other models, which were fixed at zero in Model 5, received negligible weight. Consequently, the reported BMA estimates and connectivity architecture (Figure 5B) correspond to Model 5. Figure S3 depicts the estimated parameters for fixed connections (A‐matrix) between all the ROIs. Figure S4 depicts the modulatory effects of left‐ and right‐hand movements in the averaged winning family model, showing significant interhemispheric inhibitory coupling patterns between bilateral M1 during reaching movements (Figure S4), as in previous work (Grefkes et al. 2008; Pool et al. 2014). The mean coupling parameters of the modulatory effects and their posterior probabilities are presented in Table S2.

**FIGURE 5:**
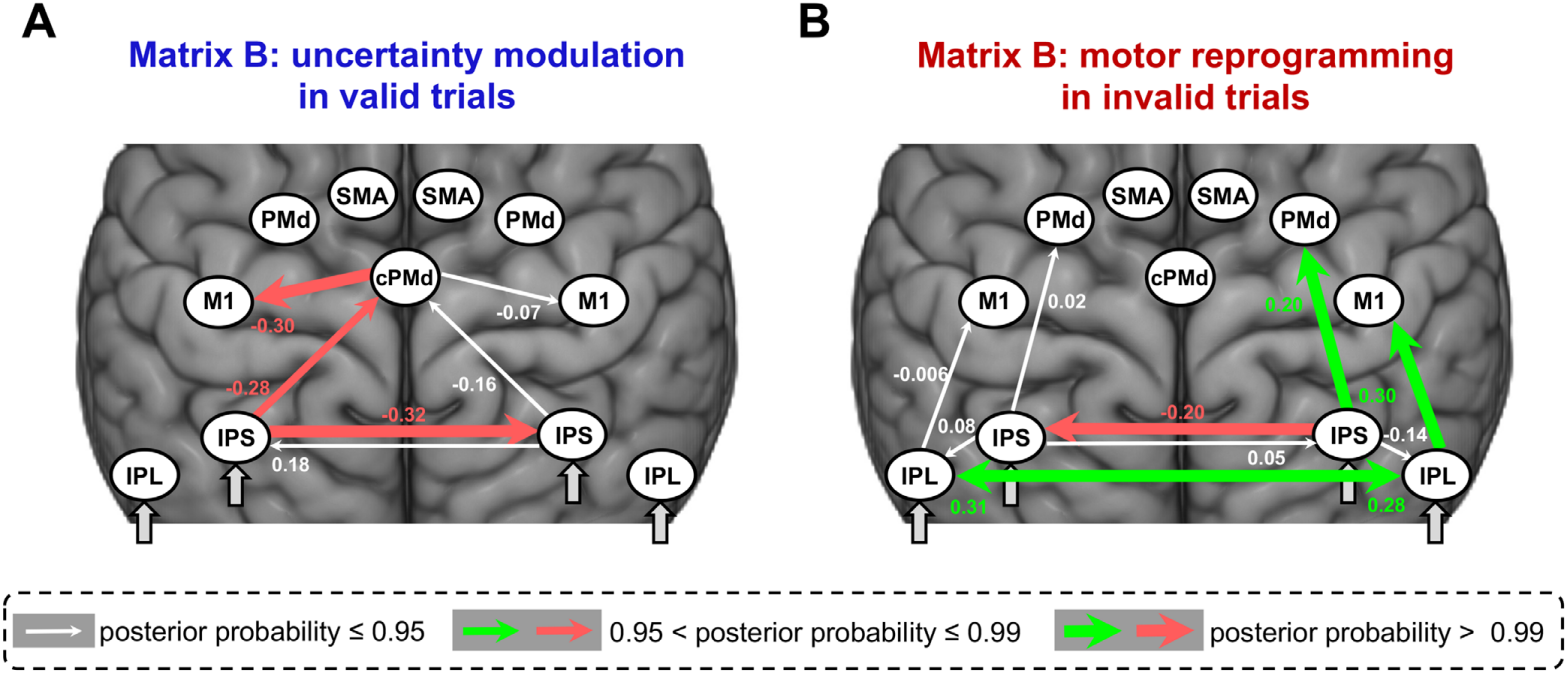
Results of Bayesian model averaging on the winning family (F1, the first modulation variant in the middle panel of Figure 4B) for the uncertainty modulation in valid trials (A) and motor reprogramming in invalid trials (B). Figure B shows connections with nonzero BMA estimates. Given that Model 5 dominated within the winning family, these connections correspond to Model 5's architecture. Green and red arrows represent positive and negative coupling parameters, respectively, with posterior probabilities of nonzero > 95%. The thicker lines indicate coupling parameters with a posterior probability > 99%, whereas the thinner lines represent those with a posterior probability > 95% but ≤ 99%. White lines denote coupling parameters with a posterior probability ≤ 95%.

Figure 5 depicts the results of the modulatory effect of uncertainty in valid trials and motor reprogramming in invalid trials on the neural coupling between the ROIs. When cue predictability about the motor response was low (uncertainty was high) in valid trials, higher coupling was observed from left IPS to cPMd. Moreover, cPMd exerted a strong negative influence on left M1, which was involved in reaching movements, also reflecting higher coupling with lower cue predictability (higher uncertainty). Similar patterns with negative connectivity parameters were observed in the right hemisphere, but with posterior probabilities below 0.95. The connection from left to right IPS was also negatively modulated by uncertainty in valid trials (Figure 5A). For the motor reprogramming subnetwork, strong positive modulations by invalid cueing were observed for connections from right IPS to movement‐related areas in right PMd, from right IPL to right M1, and between bilateral IPL (Figure 5B). Meanwhile, right IPS exerted a negative influence on left IPS during motor reprogramming.

## 4 Discussion

In the present study, we combined computational modeling of the RS of reaching movements with fMRI to demonstrate how motor control is governed by uncertainty in an extended parietal‐motor network in the human brain. Behaviorally, RS was slower in invalid than valid trials, and this difference between invalid and valid trials increased with higher cue predictability (lower uncertainty). This effect was driven by an uncertainty modulation of responses in valid trials. At the neural level, activity in bilateral IPS and cPMd was modulated by cue predictability in valid trials. Specifically, high uncertainty about target position and motor response, as indicated by low cue predictability, led to increased activity in these areas. Moreover, DCM revealed that uncertainty modulated connectivity between intraparietal and motor areas during valid trials. Lower cue predictability (higher uncertainty) increased connection strength from left IPS to cPMd, from cPMd to left M1, and from left IPS to right IPS. The uncertainty‐related connectivity modulations were more pronounced in the left hemisphere, with intraparietal‐motor connections in the right hemisphere showing similar trends but a posterior probability of nonzero below 0.95. In contrast, motor reprogramming following invalid cues showed a right‐hemispheric lateralization, with connectivity modulated between the right IPS and PMd, as well as between the right IPL and M1.

Uncertainty is a crucial factor in motor control, as actions often require selecting from a range of possibilities. Different paradigms have been used to study the role of uncertainty in sensorimotor control, such as presenting multiple possible targets or competing distractors (Arai et al. 2004; Cisek and Kalaska 2005; Tsay et al. 2021; Wong and Haith 2017). Work from these paradigms has shown that although movement plans are sometimes averaged across target locations (Chapman et al. 2010), this may occur only when slow‐speed criteria are required and the spatial separation between potential targets is low (Wong and Haith 2017). Moreover, a recent study suggested that, when faced with uncertainty, participants used the observed information to generate a motor plan that optimized task performance rather than averaging potential motor plans (Alhussein and Smith 2021). These results align with Bayesian frameworks of motor control such as active inference (Friston 2010, 2011) and are consistent with Orbán and Wolpert's (2011) proposal that the brain builds internal models to describe how environmental states give rise to observed sensory stimuli to predict how sensory inputs translate into movements.

By inducing different levels of cue predictability combined with a formal computational approach—and thereby different levels of model‐derived uncertainty about the target location and the corresponding motor response—our study provides further insights into how the brain represents and uses uncertainty to guide and optimize motor behavior. Our results show that uncertainty‐dependent processing involves flexible coupling among IPS, cPMd, and M1. These areas belong to the parietal‐premotor network, which is crucial for motor intention and control (Desmurget and Sirigu 2009). A recent study in primates has shown that specific parietal cells adjust their activity in response to levels of uncertainty by prioritizing relevant cues and efficiently using the information from those cues when making eye‐movement decisions (Horan et al. 2019). PMd neurons are involved in retrieving and combining necessary information to decide which movement to perform (Riehle and Requin 1989). Moreover, recent evidence indicates that increased uncertainty about target information modulates neural discharge in PMd and has persistent effects on reach representations during preparation and execution (Dekleva et al. 2016). The cPMd, which was modulated by uncertainty in the current study, appears to share functional roles with the PMd neurons described in the monkey studies mentioned above. In the human brain, different subregions of left and right PMd have been identified by connectivity‐based parcellations and cytoarchitectonic mapping (Genon et al. 2017, 2018; Ruland et al. 2025). According to this anatomical work, the uncertainty‐sensitive area in the present study belonged to cPMd (6d1), which is well connected with M1, SMA, and the parietal operculum—and is (among others) involved in spatial prediction, action execution, and motor learning (Genon et al. 2017, 2018; Schubotz et al. 2003).

Our results provide further insight into the interaction between the motor and intraparietal regions when reaching movements are executed under different levels of uncertainty. More specifically, the uncertainty‐dependent connectivity changes may reflect different functional roles of IPS, cPMd, and M1. Desmurget and Sirigu (2012) proposed two forms of motor intentions triggered by goal‐directed actions: the first intention—“wanting to move”—is generated by parietal areas which specify the goal to be reached before movement planning; and the second intention—“urge to move”—involves medial precentral areas and signals the moment when the planned movement is about to be initiated (Desmurget and Sirigu 2012). Transferred to our paradigm and results, it seems likely that IPS is involved in processing and integrating information from the cues and their predictive value, while cPMd may act as a coordinator, transferring relevant information from IPS to M1 to regulate movement execution in accordance with inferred uncertainty. The modulated coupling from this coordinator node to M1 may reflect the process of eliminating unlikely actions and selecting the final action as the target information becomes available. The left‐lateralized uncertainty‐related connectivity is particularly noteworthy given that left‐ and right‐hand reaching movements were balanced in this study. As all participants were right‐handed, this pattern may suggest that the dominant hemisphere maintains supervisory control over motor uncertainty, regardless of which hand executes the movement.

In contrast to the robust uncertainty modulation in valid trials, we did not observe a strong predictability‐dependent modulation of RS or neural activity in invalid trials. This is in contrast to previous studies using spatial attentional cues or cueing of right‐hand finger movements, in which neural activity in temporoparietal regions was particularly increased with higher cue predictability in invalid trials, with no or only weak modulations in valid trials (Dombert et al. 2016; Kuhns et al. 2017; Vossel et al. 2015). This may point to differences between the attention and the motor system. Moreover, previous studies did not vary the cue‐target SOA. Given that the behavioral modulation in invalid trials was modulated by SOA, this could be another factor contributing to these differences.

Still, as expected, we found generally slower responses and increased activity in bilateral PM, IPL, IPS, and temporoparietal cortex in invalid trials than in valid trials, that is, in trials requiring motor reprogramming. At the network level, motor reprogramming increased connectivity between bilateral IPL, from right IPS to right PMd, and from right IPL to right M1, and decreased connectivity from right to left IPS. These results are consistent with previous findings that motor reprogramming involves parietal and premotor regions (Petruo and Beste 2021). At first glance, the right‐lateralized connectivity during motor reprogramming in our study appears to contrast with previous TMS findings showing left hemisphere dominance for action reprogramming (Hartwigsen et al. 2012; Hartwigsen and Siebner 2015; Rushworth et al. 2001). However, this apparent contradiction likely reflects fundamental differences in the cognitive processes underlying reprogramming across different tasks. Left‐hemisphere contributions to motor reprogramming have been extensively studied using TMS targeting the left supramarginal (SMG) and premotor areas. As proposed by Mars et al. (2007), hemispheric lateralization in motor control may depend on whether reprogramming relies on arbitrary stimulus–response mappings versus spatial processing demands. Although previous TMS studies also employed a motor cueing task with validly and invalidly cued trials, participants pressed the response button with their right index finger for left‐sided targets or right middle finger for right‐sided targets, requiring action selection based on pre‐learned stimulus–response associations—a process dominated by left hemisphere networks. In contrast, our paradigm required participants to select between different hands for reaching movements toward distinct spatial targets, which involved both spatial reorienting and motor coordination. The right hemisphere may be preferentially engaged when reprogramming involves spatial reorienting of attention rather than action selection per se. This aligns with Mars et al.'s proposal that right hemisphere dominance in action reprogramming reflects spatial motor coordination demands, particularly when selecting alternative responses based on spatial target locations.

More broadly, our findings have potential implications for psychiatric and neurological disorders characterized by aberrant precision control. Within active inference accounts of motor control, the encoding of uncertainty corresponds to precision‐weighting, that is, context‐sensitive gain on prediction errors at different levels of the cortical hierarchy (Adams et al. 2013; Shipp 2016), with sensorimotor beta‐band oscillations proposed as a neurophysiological correlate of this precision‐weighting in healthy humans (Palmer et al. 2019). Disruption of this precision control has been proposed as a common mechanism across several psychiatric and neurological conditions (Limanowski 2022). Parkinson's disease provides a particularly relevant example: difficulties initiating and maintaining voluntary movement have been linked to impaired sensory attenuation and reduced precision of sensorimotor predictions (Wolpe et al. 2018), and altered movement‐related beta dynamics have been reported in patients with Parkinson's disease relative to healthy controls (Vinding et al. 2019). Our results characterize the network‐level effects of uncertainty in the motor system in healthy adults, complementing the reported electrophysiological signatures.

The connectivity model examined here is cortical, in line with our aim of characterizing the cortical motor hierarchy. Our GLM results nonetheless revealed a modulatory effect of inferred cue predictability in the cerebellum during valid trials. Both the cerebellum and the basal ganglia–thalamo‐cortical loops are strongly implicated in the prediction and revision of movement: the cerebellum is widely understood to use forward models to predict the sensory consequences of action and to compute the sensory prediction errors used to update motor commands (Shadmehr et al. 2010; Wolpert et al. 1998), while thalamic nuclei relay cerebellar and pallidal signals to premotor and motor cortex (Bostan and Strick 2018) and have been implicated in the selection and switching of prepared actions (Nakajima and Halassa 2017). In the active inference framework adopted here, these structures are natural candidates for contributing to or inheriting the precision weighting we observe in cPMd (Adams et al. 2013). We did not include the cerebellar regions in the present connectivity model to keep the model space tractable, but incorporating them, along with thalamic and basal ganglia nodes, into larger model spaces is a promising direction for future studies. A further valuable direction would be to record cardiac and respiratory signals concurrently and incorporate them into the model since motor tasks can induce small, stimulus‐locked physiological changes that contribute to the BOLD signal.

## 5 Conclusion

Taken together, the present study provides novel insights into the functional neural architecture underlying the predictive control of reaching movements. We characterized distinct interconnected functional subnetworks within a comprehensive network model of motor control: a network comprising IPS, cPMd, and M1 processing uncertainty in valid trials, and a network of intraparietal, inferior parietal, and core motor execution areas coordinating motor output and reprogramming during invalid trials.

## Author Contributions

S.V., P.M., and G.R.F. conceived the project and applied for the funding. A.Z., P.M., G.R.F., and S.V. designed the study. A.Z. performed experiments, collected data, prepared data analysis scripts, and analyzed parts of behavioral and eye movement data. S.V. analyzed parts of the behavioral and neuroimaging data. N.L. analyzed the neuroimaging data and drafted the initial manuscript. All authors reviewed and revised the manuscript.

## Funding

This work was supported by the Deutsche Forschungsgemeinschaft (Project‐ID 431549029—SFB 1451).

## Ethics Statement

This study was approved by the ethics committee of the German Psychological Society.

## Consent

All participants gave written informed consent before participating in the study.

## Conflicts of Interest

The authors declare no conflicts of interest.

## Supporting information


**Table S1:** Overview of the region of interest definition for the time series extraction.
**Table S2:** Mean B‐matrix parameters and their posterior probability of being nonzero.
**Figure S1:** (A) Mean percentage of fixation time during the cue period in all trials as a function of true %CP (left panel), and mean percentage of fixation time during the target period in valid and invalid trials as a function of %CP (right panel). (B and C) Mean RT and accuracy for each %CP in valid and invalid trials, separately for targets presented on the left and right sides, as a function of %CP. (D and E). Mean RT and accuracy for each %CP for SOAs of 800 and 1000 ms, separately in valid and invalid trials, as a function of the %CP.
**Figure S2:** Comparison of the anatomical location of cPMd and more lateral movement‐related premotor areas, along with their parameter estimates across different experimental conditions. Activity of cPMd was modulated by uncertainty in valid trials but showed no difference between reaching to left or right targets. Motor areas, including M1, PMd, and SMA, were activated during left/right reaching movements. These movement‐related areas exhibited a significant contralateral movement effect, but were not influenced by uncertainty (see parameter estimate bar charts in left/right PMd as an example).
**Figure S3:** Illustration of fixed connectivity (DCM A‐matrix) estimated by Bayesian model averaging of the winning model family. (A) Model structure. (B) Posterior A‐matrix parameters. The color of each cell represents the mean connection strength.
**Figure S4:** Illustration of the average modulatory effect of left‐ versus right‐hand movements in the winning model family.

## Data Availability

The behavioral data as well as the preprocessed imaging data and statistical maps that support the findings of this study will be openly available upon publication in the G‐Node GIN repository (https://doi.gin.g‐node.org/10.12751/g‐node.t8hbir/). The raw fMRI data will not be made publicly available due to privacy restrictions.
