# Supplementary material for: Brain Dynamics Underlying Reaching Movements Under Uncertainty
Source: Hum Brain Mapp. 2026 Oct 1;47(14):e70643. doi: 10.1002/hbm.70643 (PMC13631466; doi:10.1002/hbm.70643)
Supplement: Supplementary file 1 [file HBM-47-e70643-s001.docx]

**Supplementary Materials**

**Liu, Zabicki, Fink, Mengotti & Vossel. Brain Dynamics Underlying Reaching Movements Under Uncertainty**

As additional behavioral analyses, 2 (*validity*: valid/invalid) × 3 (*true* *%CP*: 50/70/90) × 2 (*side of target/hand*: left/right) within-subject ANOVAs on mean RTs and accuracy were performed. These revealed no significant main effect of *side/hand* (RT: *F*_(1,27)_ = 0.19, *p* = 0.67, ηₚ² = 0.01; accuracy: *F*_(1,27)_ = 1.79, *p* = 0.19, ηₚ² = 0.06; see Supplementary Figure S1B and Figure S1C), and no significant interactions of the factor *side/hand* with any of the other factors.

For the effects of cue-target SOA, a 2 (*validity*: valid/invalid) × 3 (*true* *%CP*: 50/70/90) × 2 (*SOA*: 800/1000 ms) within-subject ANOVA on mean RTs revealed significant main effects of all three factors: *validity* (*F*_(1,27)_ = 42.33, *p* < 0.001, ηₚ² = 0.61), *true* *%CP* (*F*_(2,54)_ = 3.72, *p* = 0.03, ηₚ² = 0.12), and *SOA* (*F*_(1,27)_ = 17.74, *p* < 0.001, ηₚ² = 0.40). Additionally, there was a significant *validity* × *true* *%CP* interaction (*F*_(2,54)_ = 12.65, *p* < 0.001, ηₚ² = 0.32) and a three-way interaction (*F*_(2,54)_ = 6.76, *p* = 0.002, ηₚ² = 0.20). To investigate the origin of this interaction, we performed separate 2 (*true* *%CP*: 50/70/90) × 2 (*SOA*: 800/1000 ms) ANOVAs for valid and invalid trials. In valid trials, there were significant main effects of *true* *%CP* and *SOA*, with faster responses for higher %CP and longer SOA (*F*_(2,54)_ = 14.70, *p* < 0.001, ηₚ² = 0.35; *F*_(1,27)_ = 14.08, *p* < 0.001, ηₚ² = 0.34). The interaction was not significant (*F*_(2,54)_ = 2.94, *p* = 0.06, ηₚ² = 0.10, see Supplementary Fig. S1D). In invalid trials, the main effect of *true* *%CP* was not significant (*F*_(2,54)_ = 0.10, *p* = 0.91, ηₚ² = 0.004). However, the main effect of *SOA* and the two-way interaction were significant (*F*_(1,27)_ = 8.55, *p* = 0.007, ηₚ² = 0.24; *F*_(2,54)_ = 4.19, *p* = 0.02, ηₚ² = 0.13), indicating that the effect of *true* *%CP* was modulated by *SOA* in invalid trials. This may be one of the reasons why the %CP modulation was absent in invalid trials when trials with varying SOAs were combined. Post-hoc t-tests revealed a trend towards slower responses at shorter SOA compared to longer SOA at 90% CP (*t*_(27)_ = -2.99, *p* = 0.088), and no significant differences were found at other %CP levels (see Supplementary Fig. S1D). The same 2 (*validity*: valid/invalid) × 3 (*true* *%CP*: 50/70/90) × 2 (*SOA*: 800/1000 ms) ANOVA on accuracy revealed a significant main effect of *validity* (*F*_(1,27)_ = 19.90, *p* < 0.001, ηₚ² = 0.42) and a significant *validity* × *SOA* interaction (*F*_(1,27)_ = 10.35, *p* = 0.003, ηₚ² = 0.28). Post-hoc t-tests showed that the validity effect on accuracy (valid - invalid) was significantly larger for longer SOA compared to shorter SOA (*t*_(27)_ = 3.22, *p* = 0.003, see Supplementary Figure S1E).

To confirm that the invalid > valid contrast was not confounded by differential head motion potentially caused by switching the responding hand in invalid trials, we compared framewise displacement (FD) between the two conditions. To do this, we first defined a 1.6 s window per trial, including the volume containing the target onset and the following volume. Within this window, we computed each trial's FD (averaged across the two volumes) and compared valid with invalid trials across the 28 participants with paired t-tests. The results showed that FD did not differ between valid and invalid trials (*t_(_*_27)_ = −0.03, *p* = 0.97, Cohen’s d = 0.01), indicating that the two conditions did not differ in head motion and that differential head motion is unlikely to have confounded the BOLD signal.

**Table S1. Overview over the region of interest definition for the time series extraction.**

| **ROIs** | **Definition** | **MNI Coordinates** |
| --- | --- | --- |
| M1 | *F*-contrast of valid_left vs. valid_right trials masked by Areas 4a and 4p template | -38, -22, 58; 34, -24, 56 |
| SMA | *F*-contrast of valid_left vs. valid_right trials masked by SMA template | -6, -14, 52; 8, -20, 52 |
| PMd | *F*-contrast of valid_left vs. valid_right trials masked by Area 6 template | -32, -18, 70; 30, -14, 72 |
| IPL | *t*-contrast of invalid>valid trials masked by PF&PFm template | -56, -54, 30; 64, -44, 40 |
| IPS | *t*-contrast for the negative parametric modulation effect in valid trials against zero, masked by the *t*-contrast of invalid>valid trials and by IPS template | -28, -60, 56; 30 -54 44 |
| cPMd | *t*-contrast for the negative parametric modulation effect in valid trials against zero | 2, -16, 78 |

**Table S2. Mean B-matrix parameters and their posterior probability of being non-zero.**

| Connection | Mean B-matrix parameter | Posterior probability |
| --- | --- | --- |
| *B-matrix: left hand movement* | | |
| rM1 -> lM1 | -0.265 | 0.999 |
| lM1 -> rM1 | 0.046 | 0.757 |
| rPMd -> rM1 | 0.086 | 0.894 |
| rSMA -> rM1 | 0.455 | 0.905 |
| rPMd -> lPMd | 0.064 | 0.999 |
| lPMd -> rPMd | 0.126 | 0.989 |
| rSMA -> rPMd | 0.149 | 0.996 |
| rSMA -> lSMA | -0.248 | 0.999 |
| rPMd -> rSMA | 0.216 | 0.999 |
| lSMA -> rSMA | 0.207 | 0.999 |
| *B-matrix: right hand movement* |  |  |
| rM1 -> lM1 | -0.237 | 0.999 |
| lM1 -> rM1 | -0.114 | 0.969 |
| lPMd -> lM1 | 0.497 | 0.999 |
| lSMA -> lM1 | -0.082 | 0.893 |
| lPMd -> rPMd | 0.303 | 0.999 |
| rPMd -> lPMd | -0.207 | 0.999 |
| lSMA -> lPMd | -0.064 | 0.878 |
| lSAM -> rSMA | -0.141 | 0.999 |
| lPMd -> lSMA | 0.597 | 0.999 |
| rSMA -> lSMA | 0.140 | 0.990 |
| *B-matrix: uncertainty modulation in valid trials* | | |
| cPMd -> lM1 | -0.298 | 0.999 |
| cPMd -> rM1 | -0.072 | 0.786 |
| lIPS -> cPMd | -0.280 | 0.989 |
| rIPS -> cPMd | -0.163 | 0.898 |
| rIPS -> lIPS | 0.180 | 0.947 |
| lIPS -> rIPS | -0.317 | 0.999 |
| *B-matrix: motor reprogramming in invalid trials* | | |
| lIPL -> lM1 | -0.006 | 0.545 |
| rIPL -> rM1 | 0.304 | 0.999 |
| lIPS -> lIPL | 0.078 | 0.820 |
| lIPL -> rIPL | 0.280 | 0.999 |
| rIPS -> rIPL | -0.140 | 0.939 |
| rIPL -> lIPL | 0.309 | 0.999 |
| rIPS -> lIPS | -0.200 | 0.996 |
| lIPS -> rIPS | 0.045 | 0.730 |
| lIPS -> lPMd | 0.024 | 0.683 |
| rIPS -> rPMd | 0.202 | 0.999 |

**Figure S1**. **A**. Mean percentage of fixation time during the cue period in all trials as a function of true %CP (left panel), and mean percentage of fixation time during the target period in valid and invalid trials as a function of %CP (right panel). **B-C**. Mean RT and accuracy for each %CP in valid and invalid trials, separately for targets presented on the left and right sides, as a function of the %CP. **D-E**. Mean RT and accuracy for each %CP for SOAs of 800 ms and 1000 ms, separately in valid and invalid trials, as a function of the %CP.

**Figure S2**. Comparison of the anatomical location of cPMd and more lateral movement-related premotor areas, along with their parameter estimates across different experimental conditions. Activity of cPMd was modulated by uncertainty in valid trials but showed no difference between reaching to left or right targets. Motor areas, including M1, PMd, and SMA, were activated during left/right reaching movements. These movement-related areas exhibited a significant contralateral movement effect, but were not influenced by uncertainty (see parameter estimate bar charts in left/right PMd as an example).

**Figure S3**. Illustration of fixed connectivity (DCM A-matrix) estimated by Bayesian model averaging of the winning model family. **A** Model structure. **B** Posterior A-matrix parameters. The color of each cell represents the mean connection strength.

**Figure S4**. Illustration of the average modulatory effect of left- versus right-hand movements in the winning model family.
